# Isostrictiniin Alleviates LPS-Induced Acute Lung Injury via the Regulation of the Keap1-Nrf2/HO-1 and MAPK/NF-κB Signaling Pathways

**DOI:** 10.3390/ijms26125912

**Published:** 2025-06-19

**Authors:** Wanting Ding, Yuan Sun, Wulipan Tuohudaali, Chenyang Li, Yuhan Yao, Jun Zhao

**Affiliations:** 1 School of Pharmacy, Xinjiang Medical University, Urumqi 830011, China; 1789637181@stu.xjmu.edu.cn (W.D.); sym5658@163.com (Y.S.); 13899833453@163.com (W.T.); 2 Xinjiang Key Laboratory for Uighur Medicine, Institute of Materia Medica of Xinjiang, Urumqi 830004, China; licy0609@126.com (C.L.); 17726840515@163.com (Y.Y.)

**Keywords:** isostrictiniin, *Nymphaea candida*, A549, anti-inflammatory, acute lung injury, Keap1-Nrf2/HO-1 signaling pathway, MAPK/NF-κB pathway

## Abstract

This study aimed to investigate the preventive effects of isostrictiniin (ITN) from *Nymphaea candida* against acute lung injury (ALI) through lipopolysaccharide (LPS)-induced ALI mice and LPS-induced A549 cells. Compared with the model group, ITN (50 and 100 mg/kg) significantly reduced the lung indexes, W/D rates, BALF WBC counts, and total protein contents in ALI mice (*p* < 0.05), as well as the blood neu counts (*p* < 0.01), while increasing the blood lym counts (*p* < 0.01). ITN (50 and 100 mg/kg) also markedly decreased the lung tissue TNF-α, IL-6, IL-1β, MDA, and MPO activities in ALI mice (*p* < 0.01) and enhanced the SOD and GSH levels (*p* < 0.01). Additionally, ITN (50 and 100 mg/kg) significantly improved lung histopathological damage in ALI mice. Moreover, ITN (10 and 25 µM) significantly reduced the NO, PGE2, IL-1β, IL-6, TNF-α, and MDA levels in LPS-induced A549 cells (*p* < 0.01) while significantly increasing the SOD and GSH activities (*p* < 0.01). After LPS-induced A549 cells, the Keap1, p-JNK/JNK, p-ERK1/2/ERK1/2, p-P38/P38, p-IκBα/IκBα, and p-NF-κBp65/NF-κB p65 levels were significantly upregulated (*p* < 0.05), whereas the Nrf2 and HO-1 protein expressions were downregulated (*p* < 0.05). After treatment with ITN (25 μM), the changes in these relative protein expressions in LPS-induced A549 cells were significantly reversed (*p* < 0.05). The above results indicate that ITN has a better preventive effect against ALI, and its mechanisms are related to the regulation of the Keap1-Nrf2/HO-1 and MAPK/NF-κB signaling pathways.

## 1. Introduction

Sepsis, an inflammatory response disorder resulting from bacterial infection, is associated with a mortality rate as high as 30% [1]. The lung is often the first organ affected, with acute lung injury (ALI) occurring in over 50% of sepsis patients [2]. If left untreated, ALI may progress to the more severe condition of acute respiratory distress syndrome (ARDS) [3,4]. The uncontrolled inflammatory response is the core mechanism of ALI. The initial purpose of inflammation is to resist external stimuli, but when the internal defense mechanisms are seriously damaged and the inflammation cannot be subsided in a timely manner, a significant accumulation of inflammatory cells occurs at the site of injury, leading to the release of numerous inflammatory mediators. This process can trigger a systemic “cytokine storm,” resulting in damage to the lung tissue structure and function [5,6,7,8,9].

Alveolar epithelial cells serve as a barrier to safeguard the lungs from inhalation damage, and their damage is closely associated with the pathogenesis and progression of ALI [10]. A549 is a basal epithelial cell of human lung adenocarcinoma and also a type II alveolar epithelial cell. Lipopolysaccharides (LPSs) can induce a cascade inflammatory response and oxidative stress imbalance in this cell, finally resulting in cell damage [11,12]. Excessive inflammatory reactions and oxidative stress are key factors contributing to the development of ALI [13]. LPS primarily stimulates toll-like receptor 4 (TLR4), triggering the downstream mitogen-activated protein kinase (MAPK) pathway and the nuclear factor-κB (NF-κB) signaling cascade [14,15]. The activation of the MAPK/NF-κB pathway leads to the upregulation of pro-inflammatory cytokines and excessive mucus production, thereby exacerbating inflammation and increasing lung permeability [16,17]. As a key regulator of antioxidation, Nrf2 binds with the intracellular protein Keap1. The binding energy of both is reduced under exposure to ROS. Nrf2 becomes activated and migrates to the nucleus, thereby promoting the expression of downstream antioxidant proteins, including HO-1, SOD, and GSH [18,19,20]. HO-1 inhibits the production of inflammatory cytokines, including IL-1β and TNF-α; reduces chemokine ligand-induced leukocyte aggregation in the alveolar septum; and maintains the integrity of the pulmonary vascular barrier [21,22]. Moreover, Nrf2 and NF-κBp65 showed competition for binding to the transcriptional coactivator CBP/p300 complex, so a high level of Nrf2 can inhibit the NF-κB pathway [23].

**Figure 1:**
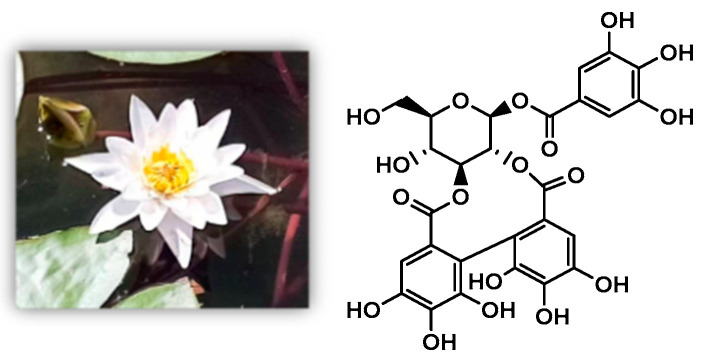
Chemical structure of ITN from *N. candida*.

Isostrictiniin (ITN, Figure 1), a polyphenol, has anti-inflammatory, antioxidant, hepatoprotective, and anti-fibrosis activities and is the primary characteristic components of the *Nymphaea candida* flower [24]. As a traditional ethnic medicine, the *N. candida* flower possesses the efficacies of moistening the lungs, alleviating cough, dissipating heat, fortifying the heart, hydrating the brain, and soothing the psyche [25]. Polyphenols are the main pharmacodynamic material base of this herb, with their content reaching up to 15.0% [26]. A previous study showed that polyphenols enriched from *N. candida* (NCTP) exhibited preventive effects against ALI. ITN, a key medicinal compound in NCTP, accounts for more than 28% of its content [27]. This study aims to elucidate the mechanisms by which ITN attenuates LPS-induced acute lung injury in a murine model and A549 cells, with a particular focus on the regulatory effects of ITN on the Keap1-Nrf2/HO-1 and MAPK/NF-κB signaling pathways. The results will provide a reference for the development and utilization of ITN as well as the determination of quality markers from *N. candida*.

## 2. Results

### 2.1. Impact of ITN on Inflammatory Responses in ALI Mice

**Figure 2:**
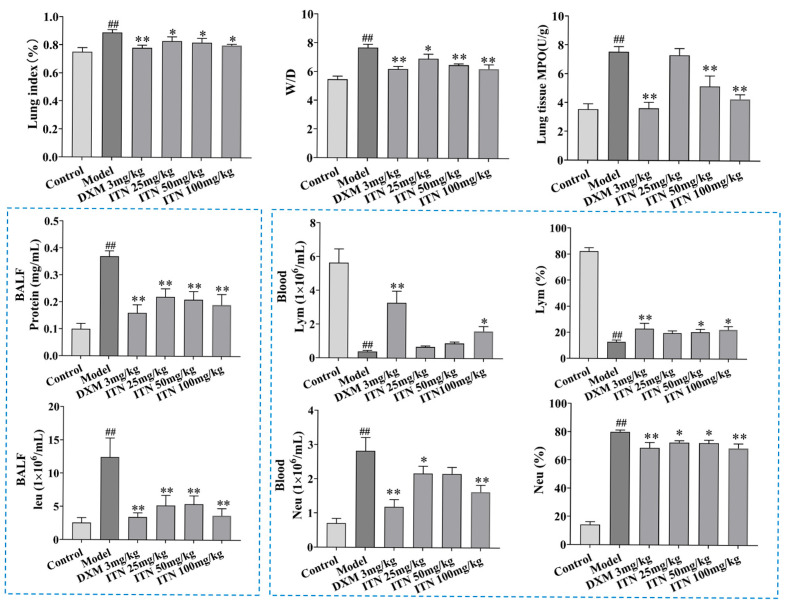
Influence of ITN on the lung index, W/D, lung tissue MPO activity, BALF total protein level, and BALF WBC counts in ALI mice, as well as blood cell (lym and neu) counts and their percentages. Data are expressed as the mean ± SEM, n = 6. ^##^ *p* < 0.01, compared with control group; * *p* < 0.05 and ** *p* < 0.01, compared with the model group.

In contrast to the control group, the following observations were made in the model mice: listlessness, slow movement, their skin lost luster, their fur was messy and not bright, their appetite and water intake decreased significantly, and their breath was rapid. The intervention of ITN (50 and 100 mg/kg) and DXM (dexamethasone, 3 mg/kg) substantially improved these states in the ALI mice, and no mice died during the experiment. As shown in Figure 2 (Table S1), the lung indexes of the ALI mice were remarkedly reduced after ITN was administered (25, 50, and 100 mg/kg, *p* < 0.05), and the lung tissue MPO activity of the ALI mice also decreased after ITN treatment (50 and 100 mg/kg, *p* < 0.05). The severity of lung edema was assessed using the lung wet-to-dry (W/D) ratio and protein leakage in bronchoalveolar lavage fluid (BALF). In the ALI mice, LPS significantly increased the W/D ratio and protein content compared to the control group (*p* < 0.05). ITN (25, 50, and 100 mg/kg) also effectively reduced protein leakage and the W/D ratio (*p* < 0.01) and significantly decreased the BALF white blood cell (WBC) counts in the ALI mice (*p* < 0.01). Moreover, ITN (100 mg/kg) also efficiently suppressed the blood neutrophil (neu) counts in the ALI mice (*p* < 0.01) and elevated the lymphocyte (lym) numbers (*p* < 0.01).

**Figure 3:**
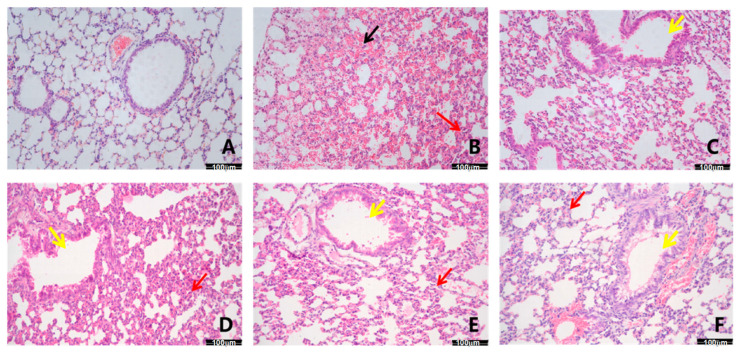
Effects of ITN on pathological tissue in ALI mice (HE, ×200). (**A**) Control; (**B**) model; (**C**) DXM, 3 mg/kg; (**D**) ITN, 25 mg/kg; (**E**) ITN, 50 mg/kg; and (**F**) ITN, 100 mg/kg. Black arrow, red blood cell exudation; red arrow, thickening of the alveolar wall; yellow arrow, degeneration and hyperplasia of the bronchial epithelium.

The mice lung tissues were stained with H&E for pathological analysis. As shown in Figure 3A,B, the mice in the control group maintained normal status, including lung structure integrity, clear alveolar structure, thin alveolar walls, and no thickening. Compared with the control group, the lung tissues in the model mice showed unique pathological changes, such as interstitial inflammatory cell infiltration, red blood cell exudation (black arrow), significant thickening of the alveolar wall (red arrow), and degeneration and hyperplasia of the bronchial epithelium (yellow arrow). After intervening with DXM (3 mg/kg) and ITN (25, 50, and 100 mg/kg), the lung tissue structure in the ALI mice appeared normal, with reduced pulmonary interstitial edema, alveolar wall thickening, and inflammation and a lower number of inflammatory cells (Figure 3C–F, Table 1). The pathological score also decreased significantly (*p* < 0.05). These results suggest that ITN had a protective effect against LPS-induced ALI in a dose-dependent manner.

**Figure 4:**
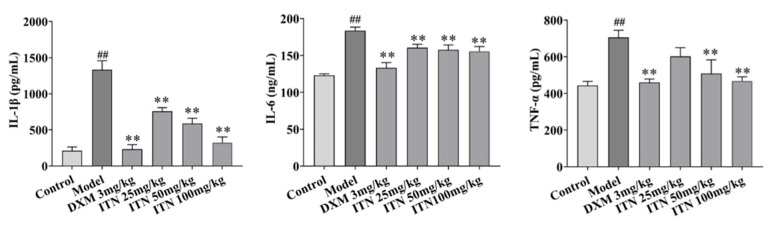
Effects of ITN on TNF-α, IL-1β, and IL-6 levels in LPS-induced mice. Data are expressed as the mean ± SEM, n = 6. ^##^ *p* < 0.01, compared with the control group; ** *p* < 0.01, compared with the model group.

The ELISA assay was performed to measure the lung tissue TNF-α, IL-1β, and IL-6 levels in the ALI mice to analyze the effects of ITN on LPS-induced inflammatory responses. The results show that ITN (50 and 100 mg/kg) effectively reduced the lung tissue TNF-α, IL-1β, and IL-6 levels in the ALI mice (*p* < 0.01, Figure 4, Table S2).

### 2.2. Effects of ITN on Lung Tissue MDA, SOD, and GSH in ALI Mice

**Figure 5:**
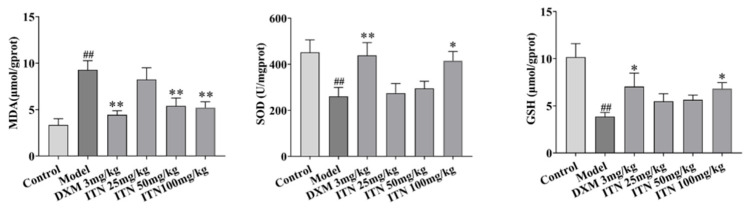
Effects of ITN on MDA, SOD, and GSH levels in LPS-induced mice. Data were expressed as mean ± SEM, n = 6. ^##^ *p* < 0.01, compared with control group; * *p* < 0.05, ** *p* < 0.01, compared with model group.

As shown in Figure 5 (Table S3), the mice lung tissue MDA levels in the model group were significantly elevated in comparison to the control group (*p* < 0.01), while the lung tissue SOD and GSH contents were considerably decreased (*p* < 0.01). Treatment with DXE (3 mg/kg) and ITN (100 mg/kg) significantly reversed these changes (*p* < 0.05), indicating that antioxidant activities are a pharmacological mechanism of ITN.

### 2.3. Cell Viability

**Figure 6:**
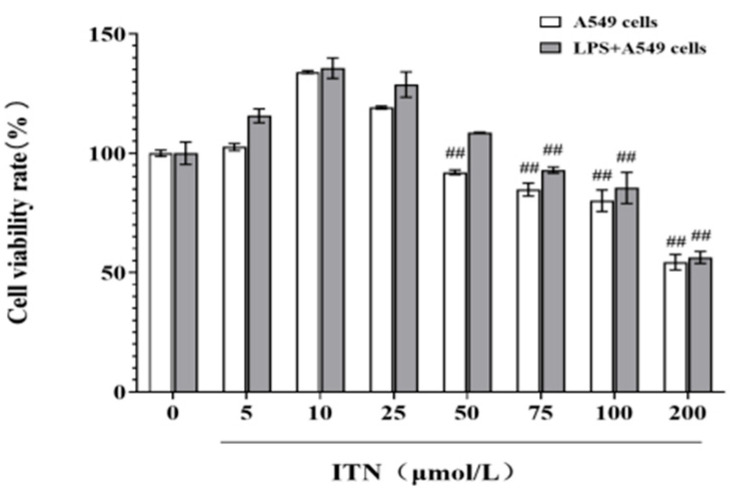
Effects of ITN on viability of A549 cells and LPS-induced A549 cells. Data are presented as mean ± SEM, n = 3. ^##^ *p* < 0.01, compared with the LPS intervention group for 12 h.

Subsequently, A549 cells and LPS-induced cells were subjected to various doses of ITN (0, 5, 10, 25, 50, 75, 100, and 200 μmol/L) for a duration of 12 h. Figure 6 illustrates that the survival rate of the ITN-treated cells at a concentration of 50 μM was markedly diminished in comparison to the control group (*p* < 0.01), signifying that ITN impeded cell proliferation at this dose, resulting in a survival rate below 80%. Consequently, an ITN concentration of 5–25 μM was employed for further tests.

### 2.4. Effects of ITN on PGE2, IL-1β, IL-6, and TNF-α Levels in A549 Cells

**Figure 7:**
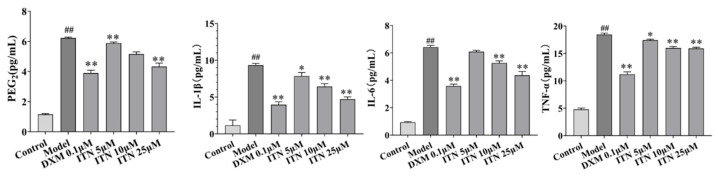
Effects of ITN on TNF-α, IL-1β and IL-6 levels in LPS irritative A549 cells. Data were expressed as mean ± SEM, n = 3. ^##^ *p* < 0.01, compared with control group; * *p* < 0.05, ** *p* < 0.01, compared with model group.

On the basis of the in vivo experiments, this study further explored the role of ITN treatment in inhibiting LPS-mediated inflammatory responses in A549 cells. As shown in Figure 7 (Table S4), in the A549 cells, LPS treatment significantly increased the pro-inflammatory IL-1β, TNF-α, and IL-6 levels (*p* < 0.05), as well as the inflammatory mediator PGE2, while ITN (25 μmol/L) significantly reduced the increases in the IL-1β, IL-6, TNF-α, and PGE2 levels induced by LPS (*p* < 0.05).

### 2.5. Effects of ITN on Oxidative Stress in A549 Cells

**Figure 8:**
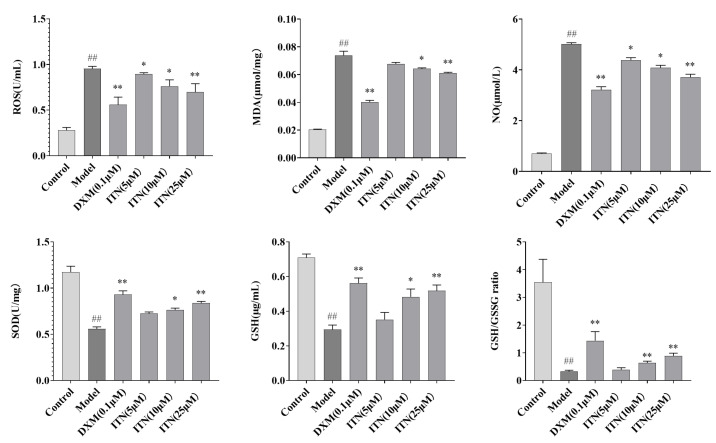
Effects of ITN on the ROS, MDA, NO, SOD, and GSH levels in LPS-induced A549 cells, as well as the GSH/GSSG ratio. Data are expressed as mean ± SEM, n = 3. ^##^ *p* < 0.01; compared with the control group; ** *p* < 0.01, * *p* < 0.05, compared with the model group,.

Figure 8 (Table S5) illustrates the impact of ITN on the concentrations of ROS, MDA, SOD, NO, and GSH in the A549 inflammatory model, as well as the GSH/GSSG ratio. In comparison to the control group, the ROS, MDA, and NO contents in the LPS group were significantly elevated (*p* < 0.01), while the SOD activity, GSH content, and GSH/GSSSG ratio were markedly reduced (*p* < 0.01). Relative to the model group, ITN (10, 25 μmol/L) exhibited a substantial reduction in the ROS, MDA, and NO levels (*p* < 0.01) and a notable increase in the SOD activity, GSH content, and GSH/GSSSG ratio (*p* < 0.01).

### 2.6. Effects of ITN on Keap1-Nrf2/HO-1 Pathway in LPS-Induced A549 Cells

**Figure 9:**
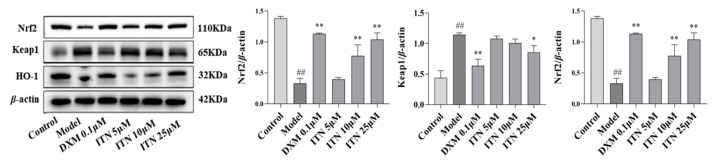
Effects of ITN on regulation of Keap1-Nrf2/HO-1 signaling pathway in LPS-induced A549 cells. Data were expressed as mean ± SEM, n = 3; ^##^ *p* < 0.01, compared with control group; * *p* < 0.05, ** *p* < 0.01, compared with model group.

As shown in Figure 9 (Figure S1), compared with the control group, the model group exhibited a significant decrease in the expression of Nrf2 and HO-1 proteins (*p* < 0.01), while Keap1 protein expression was notably elevated (*p* < 0.01). Upon treatment with ITN (10, 25 µM), the expression levels of the Nrf2 and HO-1 proteins were significantly upregulated in the LPS-induced A549 cells (*p* < 0.05). Additionally, ITN at 25 µM markedly reduced Keap1 protein expression compared to the model group (*p* < 0.05). These findings suggest that regulation of the Keap1-Nrf2/HO-1 signaling pathway may be one of the protective mechanisms of ITN against ALI.

### 2.7. Effects of ITN on MAPK/NF-κB Pathway in LPS-Induced A549 Cells

**Figure 10:**
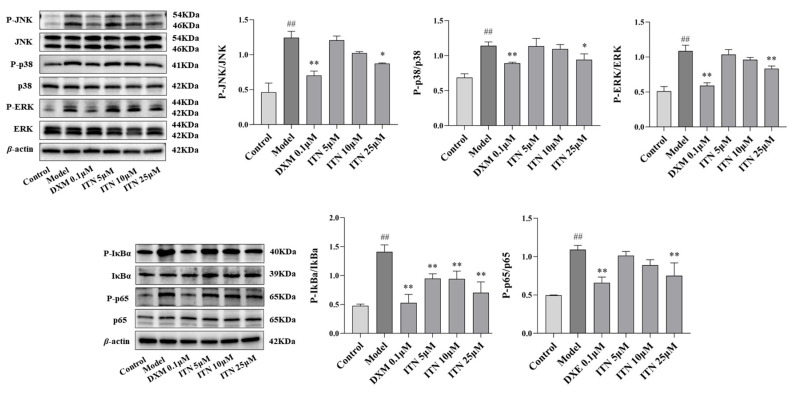
Effects of ITN on modulating MAPK/NF-κB signaling pathways in LPS-induced A549 cells. Data were expressed as mean ± SEM, n = 3; ^##^ *p* < 0.01, compared with control group; * *p* < 0.05, ** *p* < 0.01, compared with model group.

The influence of ITN on proteins related to the MAPK/NF-κB pathway was explored using an LPS-induced A549 cell injury model. As shown in Figure 10 (Figures S2 and S3), treatment with ITN (25 μM) reduced the phosphorylated protein expressions of JNK, ERK1/2, p38, IκBα, and NF-κB p65 in the LPS-induced A549 cells (*p* < 0.05). The results illustrate that ITN exerted anti-ALI activity by regulating this MAPK/NF-κB pathway.

## 3. Discussion

Sepsis is a critical illness caused by infection that leads to excessive activation of systemic inflammatory cytokines, such as TNF-α, IL-1β, and IL-6, inducing immune dysfunction and resulting in systemic organ failure. Among its complications, ARDS is the most serious. The main pathogenic mechanism involves the release of inflammatory cytokines that disrupt the balance of the body’s immune system, causing damage to alveoli and pulmonary capillaries, leading to ALI, and even respiratory failure in severe cases [28]. Therefore, the key pathological features of ALI are the destruction of the alveolar capillary barrier and an uncontrolled inflammatory response [2]. During the occurrence and development of sepsis-induced ALI, homeostasis in the alveoli is disrupted, inflammation erupts, and capillary permeability increases, leading to interstitial and alveolar edema; the immune system promotes the recruitment of phagocytic cells by producing cytokines, chemokines, and interferons, a large number of neutrophils rapidly accumulate in the lungs, releasing reactive oxygen species (ROS), proteases, and neutrophil extracellular traps, further exacerbating lung tissue damage [29,30]. LPS (also known as endotoxins), a unique component in the cell wall of Gram-negative bacteria, can activate the expression of inflammatory cytokine by binding to host cell surface receptors, cause inflammatory reactions, and initiate the pathogenic process of bacterial infection [31]. This study showed that ITN exerted preventive effects against LPS in ALI mice and LPS-induced A549 cell injury.

Oxidative stress is one of the key pathological mechanisms of ALI. MPO is a peroxidase released by neutrophils, and its overexpression can lead to the generation of oxidants, causing oxidative damage to lung tissues [32]. LPS significantly increased the MPO content in the BALF of ALI mice, but this increase was significantly reversed after treatment with ITN (50, 100 mg/kg). As a product of cell membrane lipid peroxidation, MDA has strong cytotoxicity and can lead to protein cross-linking, denaturation, and DNA breakage [33]. SOD can play an important protective role in the lungs by decomposing superoxide radicals into H_2_O_2_, thereby clearing harmful oxygen free radicals [34]. GSH can inhibit lipid peroxidation, protects cell membranes, and helps restore cellular function. Under the catalysis of glutathione peroxidase (GSH-PX), GSH can reduce H_2_O_2_ production in cells, turning it into O_2_ and H_2_O. Meanwhile, GSH is oxidized to form oxidized glutathione (GSSG), which is then catalyzed by glutathione reductase to regenerate GSH. Therefore, the GSH/GSSG ratio is one of the main indicators of the glutathione redox cycle, which can well reflect the redox state of cells [35]. In this study, ITN significantly decreased the lung tissue MDA in LPS-induced ALI mice and LPS-induced A549 cells, increasing SOD and GSH activities. Moreover, ITN could also reduce the ROS level in LPS-induced A549 cells and improve the GSH/GSSG ratio.

Pro-inflammatory factors (TNF-α, IL-1β, and IL-6) can exacerbate ALI by activating immune cells to promote disruption of the endothelial/epithelial barrier and recruiting neutrophils [36]. TNF-α can lead to pulmonary tissue and interstitial edema by upregulating the expression of inflammatory factors, enhancing alveolar capillary permeability, and accelerating the infiltration of white blood cells in lung tissue and alveoli [37]. IL-6 promotes the infiltration of macrophages in the local bronchial mucosal epithelium and further promotes damage to CD4+ T lymphocytes in the glands and bronchial mucosa. In addition, excessive release of IL-6 can affect innate immunity, promote platelet activation, cause hemodynamic disorders, cause damage to pulmonary vascular endothelium, and exacerbate pulmonary inflammation [38]. ITN significantly decreased the TNF-α, IL-1β, and IL-6 levels in LPS-induced ALI in mice and LPS-induced A549 cells. Moreover, the pathological increase in ROS caused by LPS can further exacerbate the inflammatory response [39]. As transcription factors enhance antioxidant stress response, activation of the Nrf2 protein can significantly diminish inflammatory responses triggered by LPS, reduce neutrophil migration, and impede the secretion of inflammatory mediators by alveolar epithelial cells, thereby alleviating ALI [40]. In this experiment, ITN may have stimulated the synthesis of lung tissue Nrf2 and HO-1 proteins in the LPS-induced A549 cells and reduced keap1 protein expression, consequently enhancing the SOD and GSH activities and diminishing the MDA levels. Our results indicate that ITN alleviated LPS-induced A549 cell injury by mitigating oxidative stress damage and enhancing the repair of the alveolar–capillary barrier.

The MAPK family serves as crucial mediators of responses of inflammation to diverse signals, primarily comprising the ERK, JNK, and p38 MAPK pathways [41]. ERK can promote the proliferation and repair of alveolar epithelial cells; JNK can phosphorylate Bcl-2/Bax family proteins to induce cell apoptosis; P38 can activate macrophages and neutrophils and increase the expressions of TNF-α, IL-1β, and IL-6 [42,43]. MAPK, especially JNK, can also inhibit the Nrf2 pathway, intensifying oxidative damage; P38 and JNK can promote the nuclear translocation of NF-κB by activating IKK, amplifying the inflammatory response [44,45]. NF-κB remains inactive in the mitochondria by binding to its inhibitor protein IκB during the quiescent period. When cells are stimulated, IκB phosphorylation and NF-κB activation translocate from the cytoplasm to the nucleus, regulating downstream gene transcription [45,46]. The activation of the NF-κB pathway leads to the overexpression of pro-inflammatory cytokines, including IL-1β, IL-6, and TNF-α [47]. In this study, ITN significantly inhibited the kinase activities of p38, JNK, ERK1/2, p65, and IκBα in LPS-induced A549 cells, thereby attenuating inflammation. The results show that ITN may alleviate inflammatory damage in A549 cells by inhibiting the MAPK and NF-κB pathways.

## 4. Materials and Methods

### 4.1. Chemicals and Reagents

The human non-small cell lung cancer epithelial cell A549 (CL-0016) was purchased from Wuhan Punosei Life Technology (Wuhan, China); LPS (*Escherichia coli* O55: B5 lipopolysaccharide, L2880-100mg) was purchased from the Sigma Company in St. Louis, MO, USA. Dexamethasone (DXM; D8040), RIPA buffer (R0010), PMSF (P0100) and BCA protein concentration determination kit (PC0020) were purchased from Beijing Solebao Biotechnology (Beijing, China). Ultra-sensitive ECL chemiluminescence substrate (BL520A) was purchased from Beijing Langeco Technology (Beijing, China). The DMEM high-sugar medium (8122292) was purchased from Thermo Fisher Scientific, Waltham, MA, USA. In this study, the ELISA kits PGE2 (0034), NO (H1846), TNF-α (H0109), IL-6 (H6156), and IL-1β (H0149) were purchased from Wuhan Eliret Biotechnology (Wuhan, China). A lipid oxidation (MDA) test kit (S0131S), a total SOD activity test kit (S0101S), and a GSH and GSSG test kit (S0053) were purchased from Beyotime Biotechnology (Shanghai, China). The protein prestain marker (26616) was purchased from Thermo Fisher Scientific. The 10× TBST buffer (T1082) was purchased from Beijing Solaibao Technology (Beijing, China). Skim milk powder (1172GR500) was purchased from Guangzhou Saiguo Biotechnology (Guangzhou, China). The Keap1 antibody (ab227828), HO-1 antibody (ab52947), p-P38 antibody (ab195049), JNK antibody (ab179461), p38 antibody (ab170099), ERK1/2 antibody (ab184699), p-JNK antibody (ab76572), anti-rabbit IgG HL antibody (ab205179), and p-IκBα antibody (ab133462) were purchased from Abcam (Cambridge, UK). The p-ERK1/2 antibody (4376T), p-NF-κB p65 antibody (3033T), NF-κB p65 antibody (8242T), Nrf2 antibody (12721), IκBα antibody (4814T), TLR4 antibody (14358), and β-actin antibody (8457) were purchased from Cell Signaling Technology (Beverly, CA, USA).

### 4.2. Plant Materials and Preparation of NCTP

*N. candida* flowers were obtained from Xinjiang Shengxiangcao Co. (Urumqi, China) and identified by Researcher Jiang He at the Xinjiang Institute of Materia Medica. As per the preparation methods and detection conditions reported earlier, the N. candida flowers were extracted with 70% ethanol under reflux to obtain an extract. Subsequently, the extract was purified using D101 resin combined with polyamide to obtain the total polyphenols. Then, the total polyphenols were repeatedly purified using an MCI gel CHP-20p column to obtain ITN, and the purity of the ITN was determined as 95.97% using an HPLC normalization assay.

### 4.3. Animal Experiment

#### 4.3.1. Model Design

Kunming mice (weight of 18–20 g) were obtained from Xinjiang Medical University (Urumqi, China). All mice were kept in a controlled environment at an ambient temperature of 24 ± 1 °C and humidity at 60 ± 5%, with a 12 h light/dark cycle. The mice had free access to food and water, and they were acclimated to the environment for one week. All the animal experiments were performed in accordance with the Animal Welfare and Research Ethics Committee at Xinjiang Medical University (IACUC-20220314-2).

The mice were randomly divided into six groups (n = 10): the normal group, the model group, the DXM (3.0 mg/kg) group, and the ITN (25, 50, and 100 mg/kg) groups. The normal control group and the model group were orally given a 0.5% CMC-Na solution in the same volume, while the other mice received intragastric administration at the set dose once a day for 7 days. At 1 h after the last administration, 10 mg/kg LPS was intraperitoneally injected into the model group and the administration group, in addition to a 0.9% sodium chloride solution at the same volume in the normal control group. The body weight, water intake, food intake, appearance, behavior, secretion, and excrement of the mice were observed and recorded during the experiment. At 12 h after modeling, blood was taken from the eyeballs, and the mice were sacrificed. Routine blood tests were carried out with an animal-specific fully automatic blood cell analyzer (BC-5000VET, Shenzhen Mindray, China), including the neutrophil (neu) counts and lymphocyte (lym) counts. The lung tissue was dissected and removed, and the lung index was calculated as follows: lung index = (lung weight/body weight) g × 100%.

#### 4.3.2. Lung Wet-to-Dry Ratio (W/D Ratio)

Fresh tissue from the upper lobe of the left lung was taken, the surface was wiped dry, and then, it was weighed to obtain the wet weight. Then, it was dried in a drying oven at 80 °C for 48 h and weighed again to obtain the dry weight. Finally, the ratio of wet weight to dry weight (W/D) was calculated.

#### 4.3.3. Cell Counts and Protein Contents of Bronchoalveolar Lavage Fluid (BALF) in Mice

The mice lung tissues were rinsed with 1.0 mL of the PBS solution three times, and the bronchoalveolar lavage fluid (BALF) was obtained. The BALF was centrifuged at 4 °C at 3000 rpm for 10 min, and the supernatant was preserved for total protein content determination using a BCA assay. The precipitates were re-suspended in a PBS solution, and the animal-specific fully automatic blood cell analyzer was used to detect WBC counts.

#### 4.3.4. Lung Tissue Pro-Inflammatory Cytokines and Oxidative Stress Indicators in Mice

The lung tissue samples were homogenized in normal saline to obtain a 10% (*w*/*v*) lung homogenate and then centrifuged at 3000 rpm for 10 min at 4 °C. The supernatant was used to determine the indicators. IL-1β, IL-6, and TNF-α activities were quantified through an ELISA assay; myeloperoxidase (MPO), superoxide dismutase (SOD), reduced glutathione (GSH), and malondialdehyde (MDA) levels were determined according to the kit’s instructions.

#### 4.3.5. Histopathological Analysis

The lung tissues were fixed in a 4% paraformaldehyde solution and treated with paraffin embedding, then cut into slices about 5 μm thick, dewaxed with xylene, and dehydrated with gradient ethanol. Hematoxylin–eosin staining was performed according to the standard procedure, followed by cleaning, dehydration, and sealing. Finally, histopathological changes were observed under an optical microscope. The pathological grading was as follows: lung tissues were normal (-), less than 25% damaged (+), 25%~50% damaged (++), and more than 50% damaged (+++).

### 4.4. Cell Experiments

#### 4.4.1. Cell Culture and Treatment

Human non-small cell lung cancer cells (A549 and CL-0016) were obtained from Wuhan Punosai Life Science and Technology Co. (Wuhan, China). These cells were cultured in the RPMI-1640 medium supplemented with 10% fetal bovine serum (FBS) and antibiotics (100 µg/mL penicillin and streptomycin) at 37 °C in a humidified atmosphere containing 5% CO_2_. To examine the effects of LPS on A549 cell metabolism and proliferation, the cells were grown to confluence and then inoculated into 6-well plates following the established protocol [48]. They were subsequently randomized to a control population and the LPS group (1.0 μg/mL), with stimulation durations of 6, 12, and 24 h. The normal cells were grown in an A549 cell medium. Cells in the model group were grown in A549 cell media with a final dose of 1.0 μg/mL LPS. Cells in the exponential phase of development were selected, and the A549 cells were inoculated with a 6-well cell culture plate at a count of 7 × 10^5^ cells for each well. The procedure was carried out in accordance with the aforementioned categories. After the intervention, cell lysis was performed, followed by RNA extraction. To determine the optimal induction time for the A549 inflammatory model, the expression levels of TNF-α, IL-1β, and IL-6 in A549 cells treated with LPS were evaluated using RT-qPCR.

#### 4.4.2. CCK-8 Assay

A549 cells were seeded in a 96-well culture plate, with the cell concentration adjusted to 7 × 10^5^ cells/mL (100 μL per well), and incubated at 37 °C with 5% CO_2_ for 24 h. Following cell adhesion, the culture supernatant was removed, and fresh medium containing ITN (0, 5, 10, 25, 50, 75, 100, and 200 μmol/L) or LPS + ITN (0~200 μmol/L) was added. The cultures were then maintained for additional periods of 12, 24, and 48 h. Subsequently, the CCK-8 reagent (10 μL per well) was introduced to all experimental groups and incubated for an additional duration of two and a half hours. The survival rate of A549 cells was assessed to determine the optimal dosage and exposure time. Optical density readings were taken at a wavelength of 450 nm using a microplate reader.

#### 4.4.3. Pro-Inflammatory Cytokines and Oxidative Stress Indicators in A549 Cells

A549 cells were cultivated in 6-well plates (7 × 10^5^ cells/mL) in a 5% CO_2_ incubator at 37 °C. Upon reaching 70% confluence, the cells were administered 1 μg/mL LPS for a duration of 12 h. ITN (5.0, 10.0, and 25.0 μmol/L) and DXM (10 μM) were administered for an additional 12 h, alongside 1 μg/mL LPS. After the intervention, the cell supernatant was harvested and centrifuged, and the IL-1β, IL-6, and TNF-α levels were quantified using an ELISA assay. The reactive oxygen species (ROS), malondialdehyde (MDA), nitric oxide (NO), superoxide dismutase (SOD), reduced glutathione (GSH), and oxidized glutathione (GSSG) levels were identified in complete compliance with the kit’s instructions.

#### 4.4.4. Western Blot Analysis

A549 cells were treated as per the “Section 4.4.3” method. Protein was extracted, and the concentration was quantified following a BCA protein assay kit. Following the addition of the appropriate volume of 5× loading buffer and PBS, the proteins in each group were denatured at 100 °C for 10 min and subsequently kept at −20 °C. The protein was transferred to a PVDF membrane via SDS-PAGE electrophoresis using a 10 µg loading sample. After washing the PVDF membrane with 1× TBST at four intervals of five minutes each, the membrane was incubated in 5% skim milk powder at room temperature for two hours. Primary antibodies (Nrf2, Keap1, HO-1, p38, p-p38, JNK, p-JNK, ERK1/2, p-ERK1/2, IκBα, p-IκBα, NF-κB p65, p-NF-κB p65, and β-actin) were incubated overnight on a shaking platform at 4 °C. On the second day, following four washes with 1× TBST for 5 min each, goat anti-rabbit IgG was incubated at room temperature in the absence of light for 1.5 h (a dilution ratio of 1:10,000) and subsequently developed using an ECL luminescence kit. The target protein bands were analyzed using the ImageJ software, with β-actin serving as the internal reference.

### 4.5. Statistical Analysis

The data are presented as the mean ± SEM. Statistical comparisons between two groups were analyzed using the t-test, while differences among multiple groups were analyzed using one-way analysis of variance (ANOVA). The Kruskal–Wallis rank sum test, a non-parametric statistical method, was designed to analyze the graded data. The band gray value analysis was performed using ImageJ 1.8.0 (National Institutes of Health, NIH, Bethesda, MD, USA). GraphPad software version 8.0 (Prism, La Jolla, CA, USA) was utilized for statistical analysis. A *p* value of less than 0.05 was considered statistically significant.

## 5. Conclusions

In this study, ITN exhibited promising preventive effects against ALI in LPS-induced mouse ALI and LPS-induced A549 cell injury models, and its mechanisms are related to the regulation of the Keap1-Nrf2/HO-1 and MAPK/NF-κB signaling pathways. These findings could yield valuable information for the in-depth development of ITN, as well as *N. candida.*

## Figures and Tables

**Table 1:** Effects of ITN on the degree of lung tissue lesions in mice.

Group	Grades				Analysis
	-	+	++	+++	
Control	5	1	0	0	0.1667 ± 0.167
Model	0	0	4	2	2.3333 ± 0.211 ^##^
DXM, 3 mg/kg	0	5	1	0	1.1667 ± 0.167 **
ITN, 25 mg/kg	0	4	2	0	1.3333 ± 0.211 **
ITN, 50 mg/kg	0	4	2	0	1.3333 ± 0.211 **
ITN, 100 mg/kg	0	5	1	0	1.1667 ± 0.167 **

Data are expressed as the mean ± SEM, n = 6. ^##^ *p* < 0.01, compared with the control group; ** *p* < 0.01, compared with the model group.

## Data Availability

The data is contained within the article and Supplementary Materials.

## Supplementary Materials

The following supporting information can be downloaded at: https://www.mdpi.com/article/10.3390/ijms26125912/s1.

## Abbreviations

The following abbreviations are used in this manuscript:

ITN	isostrictiniin
NCTP	polyphenol-enriched fraction from *Nymphaea candida*
ALI	acute lung injury
LPS	lipopolysaccharide
DXM	dexamethasone
BALF	bronchoalveolar lavage fluid
Neu	neutrophil
Lym	lymphocyte
WBC	while blood cell
MPO	myeloperoxidase
IL-1β	interleukin-1β
IL-6	interleukin-6
TNF-α	tumor necrosis factor-α
NO	nitric oxide
PGE2	prostaglandin-2
ROS	reactive oxygen species
MDA	malondialdehyde
SOD	superoxide dismutase
GSH	glutathione
GSSG	oxidized glutathione
ELISA	enzyme-linked immunosorbent assay
MAPK	mitogen-activated protein kinase
JNK	c-Jun N-terminal kinase
ERK	extracellular regulated protein kinases
NF-κB	nuclear factor kappa
IκBα	inhibitor of NF-κB
Keap1	kelch-like ECH-associated protein 1
HO-1	heme oxygenase-1
SEM	standard error of mean
ANOVA	analysis of variance
