# Supplementary material for: Isostrictiniin Alleviates LPS-Induced Acute Lung Injury via the Regulation of the Keap1-Nrf2/HO-1 and MAPK/NF-κB Signaling Pathways
Source: Int J Mol Sci. 2025 Jun 19;26(12):5912. doi: 10.3390/ijms26125912 (PMC12193549; doi:10.3390/ijms26125912)
Supplement: Supplementary file 1 [file ijms-26-05912-s001.zip › ijms-3612498-supplementary.pdf]

Supplementary Materials

This supplementary materials file contains the following contents:

Supplementary Tables	Tables S1 to S5
Supplementary Figures	Figures S1 to S3

Table S1. Influence of ITN on lung index, W/D, lung tissue MPO activity, BALF total protein level and BALF WBC count in ALI mice as well as blood cells (lymphocyte, neutrophil) counts and their percentages.

Group	Lung index (%)	W/D	Lung tissue MPO(U/g)	BALF Protein (mg/mL)	BALF WBC (1× 10^6/mL)	Blood Lym (1× 10^6/mL)	Lym (%)	Blood Neu (1× 10^6/mL)	Neu (%)
Control	0.75±0.03	5.46±0.22	3.54±0.37	0.1±0.02	2.57±0.74	5.64±0.82	82.33±2.6	0.7±0.14	14.22±2.01
Model	0.89±0.02$^{\#\#}$	7.68±0.23$^{\#\#}$	3.54±0.37$^{\#\#}$	0.37±0.02$^{\#\#}$	12.44±2.85$^{\#\#}$	0.39±0.06$^{\#\#}$	12.92±1.33$^{\#\#}$	2.82±0.39$^{\#\#}$	80.02±1.29$^{\#\#}$
DXM, 3mg/kg	0.78±0.02**	6.2±0.18**	3.62±0.42**	0.16±0.03**	3.44±0.63**	3.27±0.7**	22.93±4.18**	1.19±0.21**	68.77±3.74**
ITN, 25mg/kg	0.83±0.03*	6.93±0.31*	7.32±0.46	0.22±0.03**	5.22±1.49**	0.67±0.06	19.83±1.65	2.17±0.21*	72.63±1.29*
ITN, 50mg/kg	0.82±0.03*	6.49±0.09**	5.17±0.72**	0.21±0.03**	5.42±1.24**	0.89±0.08	20.48±2.25*	2.16±0.19	72.33±2.04*
ITN, 100mg/kg	0.8±0.01*	6.23±0.29**	4.28±0.32**	0.19±0.04**	3.7±1.05**	1.61±0.29*	22.38±2.54*	1.63±0.2**	68.63±3.22**

Data were expressed as mean ± SEM, n=6. $^{\#\#}P<0.01$, compared with control group; $^{*}P<0.05$, $^{**}P<0.01$, compared with model group.

Table S2. Effects of ITN on IL-1β, IL-6 and TNF-α levels in LPS induced mice.

Group	IL-1β (pg/mL)	IL-6 (pg/mL)	TNF-α (pg/mL)
Control	211.05±52.25	122.93±2.21	443.93±22.01
Model	1334.53±122.79$^{\#\#}$	183.65±4.69$^{\#\#}$	705.56±39.19$^{\#\#}$
DXM, DXM 3mg/kg	232.11±63.54**	133.35±6.92**	459.32±19.25**
ITN, 25mg/kg	757.43±50.49**	160.45±4.7**	602.23±47.5
ITN, 50mg/kg	587.35±74.11**	157.74±6.52**	509.25±74.16**
ITN, 100mg/kg	324.21±78.34**	155.51±6.54**	467.59±23.18**

Data were expressed as mean±SEM, n=6. $^{\#\#}P < 0.01$, compared with control group; $^{**}P < 0.01$, compared with model group.

Table S3. Effects of ITN on MDA, SOD and GSH levels in LPS induced mice.

Group	MDA (μmol/gprot)	SOD(U/mgprot)	GSH (μmol/gprot)
Control	3.36±0.67	451.98±54.02	10.17±1.43
Model	9.27±1$^{\#\#}$	260.98±38.17$^{\#\#}$	3.86±0.43$^{\#\#}$
DXM, DXM 3mg/kg	4.45±0.43**	438.95±54.8**	7.05±1.42*
ITN, 25mg/kg	8.23±1.26	274.74±41.9	5.49±0.79
ITN, 50mg/kg	5.41±0.83**	295±31.08	5.64±0.5
ITN, 100mg/kg	5.21±0.64**	414.9±40.68*	6.82±0.64*

Data were expressed as mean±SEM, n=6. $^{\#\#}P < 0.01$, compared with control group; $^{*}P < 0.05$, $^{**}P < 0.01$, compared with model group.

Table S4. Effects of ITN on PGE 2, IL-1β, IL-6, and TNF-α levels in LPS irritative A549 cells.

Group	PEG2(pg/ml)	IL-1β(pg/ml)	IL-6(pg/ml)	TNF-α(pg/ml)
Control	1.154±0.0285	1.159±0.4144	0.938±0.0287	4.772±0.1503
Model	6.224±0.0322##	9.337±0.1349##	6.419±0.0761##	18.43±0.1615##
DXM,0.1μmol/L	3.898±0.1082**	3.946±0.2325**	3.592±0.0753**	11.18±0.2631**
ITN,5μmol/L	5.887±0.0389	7.860±0.2694*	6.093±0.0575	17.41±0.1061*
ITN,10μmol/L	5.161±0.0809	6.435±0.2217**	5.266±0.0890**	15.99±0.1584**
ITN,25μmol/L	4.326±0.1320**	4.707±0.1759**	4.364±0.1602**	15.90±0.1304**

Data were expressed as mean±SEM, n=3. ## $P < 0.01$, compared with control group; * $P < 0.05$, ** $P < 0.01$, compared with model group.

Table S5. Effect of ITN on the MDA, SOD, GSH, GSSG, GSH/GSSG, NO and ROS levels in LPS induced A549 cells.

Group	MDA(μmol/mg)	SOD（U/mg）	GSH(μg/mL)	GSSG(μg/mL)	GSH/GSSG	NO(μmol/L)	ROS(U/mL)
Control	0.0203 ±0.000341	1.1730 ±0.0633	0.0649 ±0.00357	0.2056 ±0.0379	3.545 ±0.8249	0.6981 ±0.031	0.2792 ±0.0315
Model	0.0738 ±0.003010##	0.5573 ±0.0231##	0.0411 ±0.00092##	0.9056 ±0.0380##	0.3251 ±0.0462##	5.016 ±0.0546##	0.9550 ±0.0247##
DXM,0.1μmol/L	0.0402 ±0.001220**	0.9302 ±0.0402**	0.0572 ±0.00121**	0.4017 ±0.0708**	1.437 ±0.3296**	3.217 ±0.1189**	0.5607 ±0.0827**
ITN,5μmol/L	0.0675 ±0.001230	0.7240 ±0.0181	0.0468 ±0.00088	0.9052 ±0.0407	0.3876 ±0.0720	4.384 ±0.0951*	0.8952 ±0.0145*
ITN,10μmol/L	0.0642 ±0.000593*	0.7641 ±0.0190*	0.05100 ±0.00104*	0.7548 ±0.0547*	0.6366 ±0.0597**	4.078 ±0.1027*	0.7625 ±0.0700*
ITN,25μmol/L	0.0610 ±0.000587**	0.8381 ±0.0179**	0.0555 ±0.00316**	0.5848 ±0.0358**	0.8871 ±0.0994**	3.705 ±0.1239**	0.6974 ±0.0925**

Data were expressed as mean±SEM, n=3. ## $P < 0.01$, compared with control group; * $P < 0.05$, ** $P < 0.01$, compared with model group.

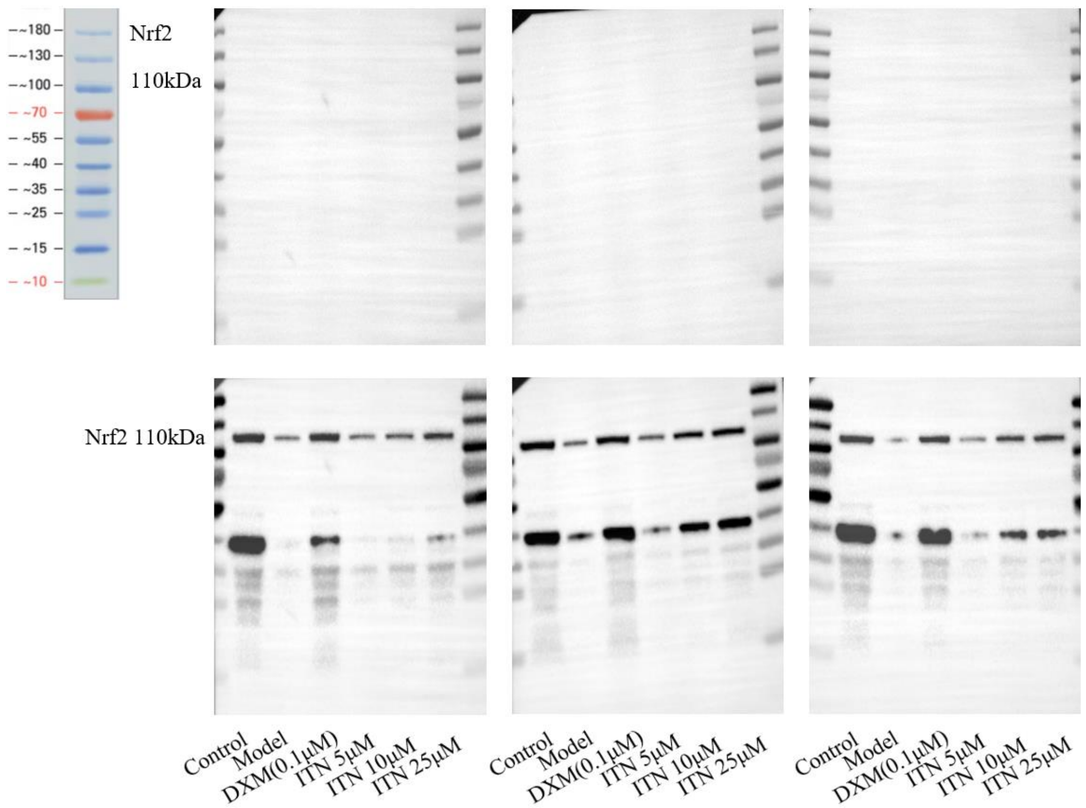
~180
~130
~100
~70
~55
~40
~35
~25
~15
~10
Nrf2
110kDa
Nrf2 110kDa
Control
Model
DXM(0.1μM)
ITN 5μM
ITN 10μM
ITN 25μM
Control
Model
DXM(0.1μM)
ITN 5μM
ITN 10μM
ITN 25μM
Control
Model
DXM(0.1μM)
ITN 5μM
ITN 10μM
ITN 25μM

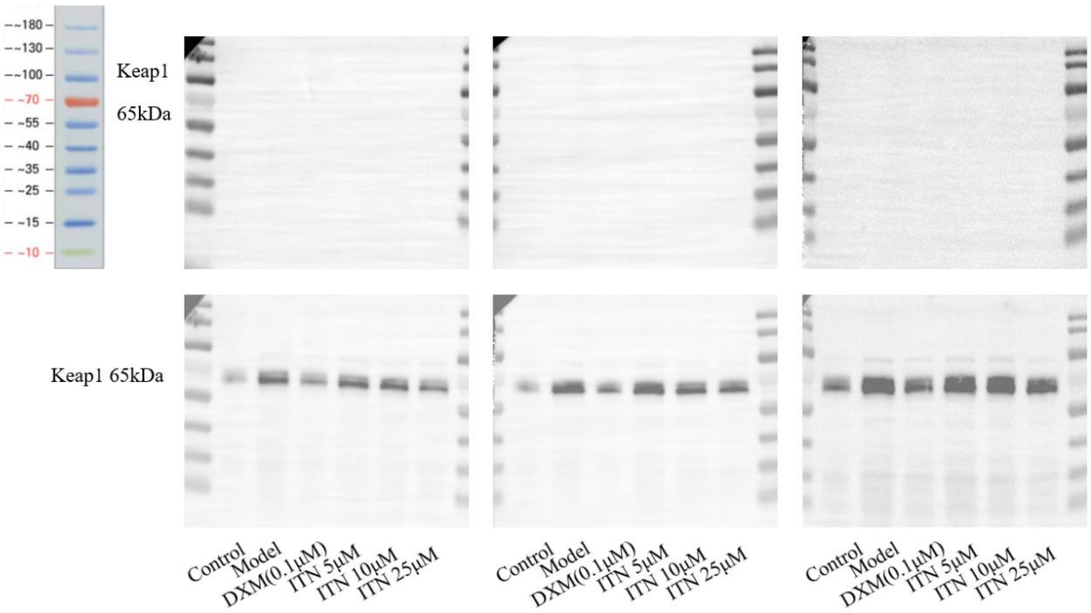
~180
~130
~100
~70
~55
~40
~35
~25
~15
~10
Keap1
65kDa
Keap1 65kDa
Control
Model
DXM(0.1μM)
ITN 5μM
ITN 10μM
ITN 25μM
Control
Model
DXM(0.1μM)
ITN 5μM
ITN 10μM
ITN 25μM
Control
Model
DXM(0.1μM)
ITN 5μM
ITN 10μM
ITN 25μM

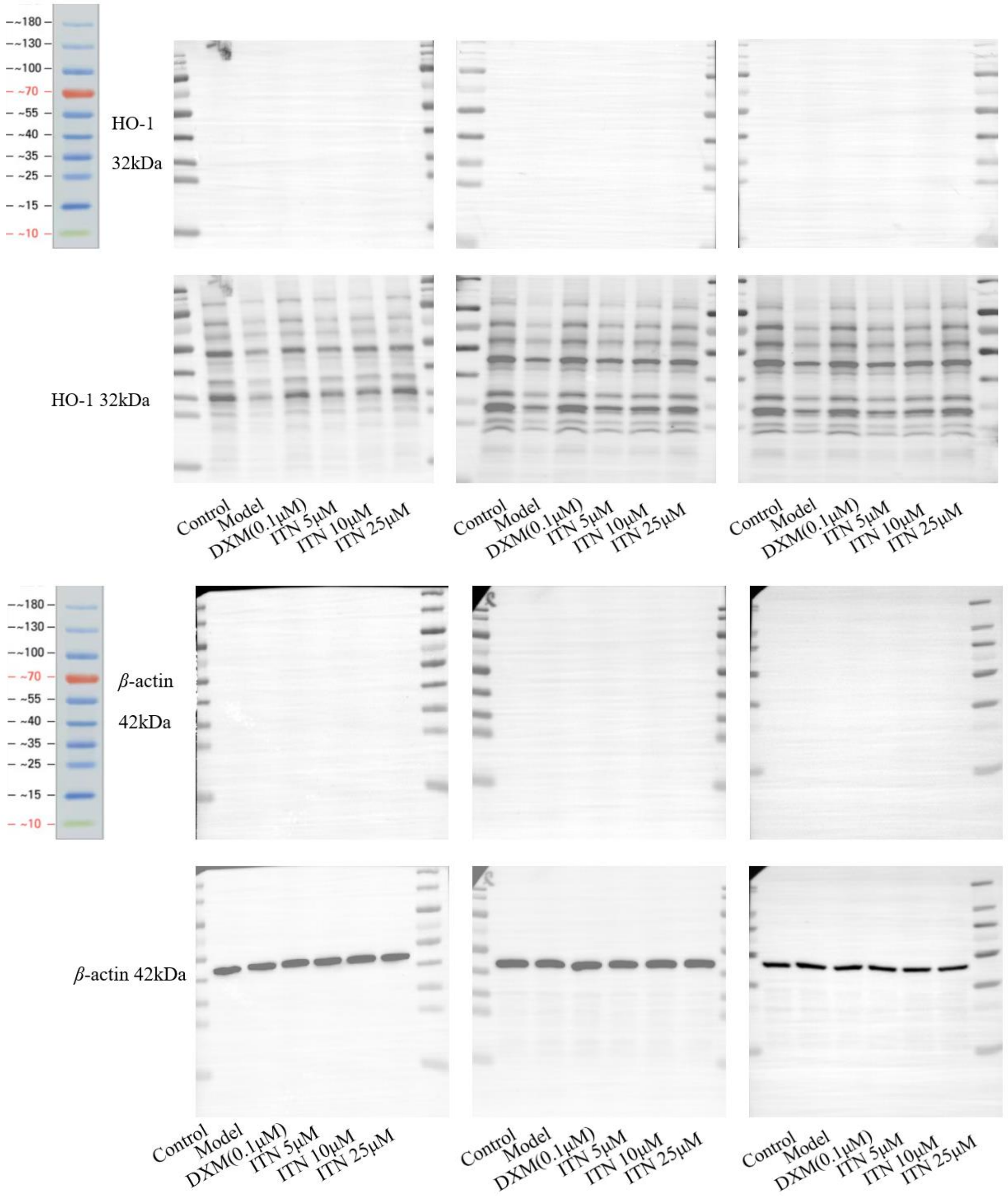

Figure S1. Effects of ITN on Keap1-Nrf2/HO-1 pathway in LPS irritated A549 cells

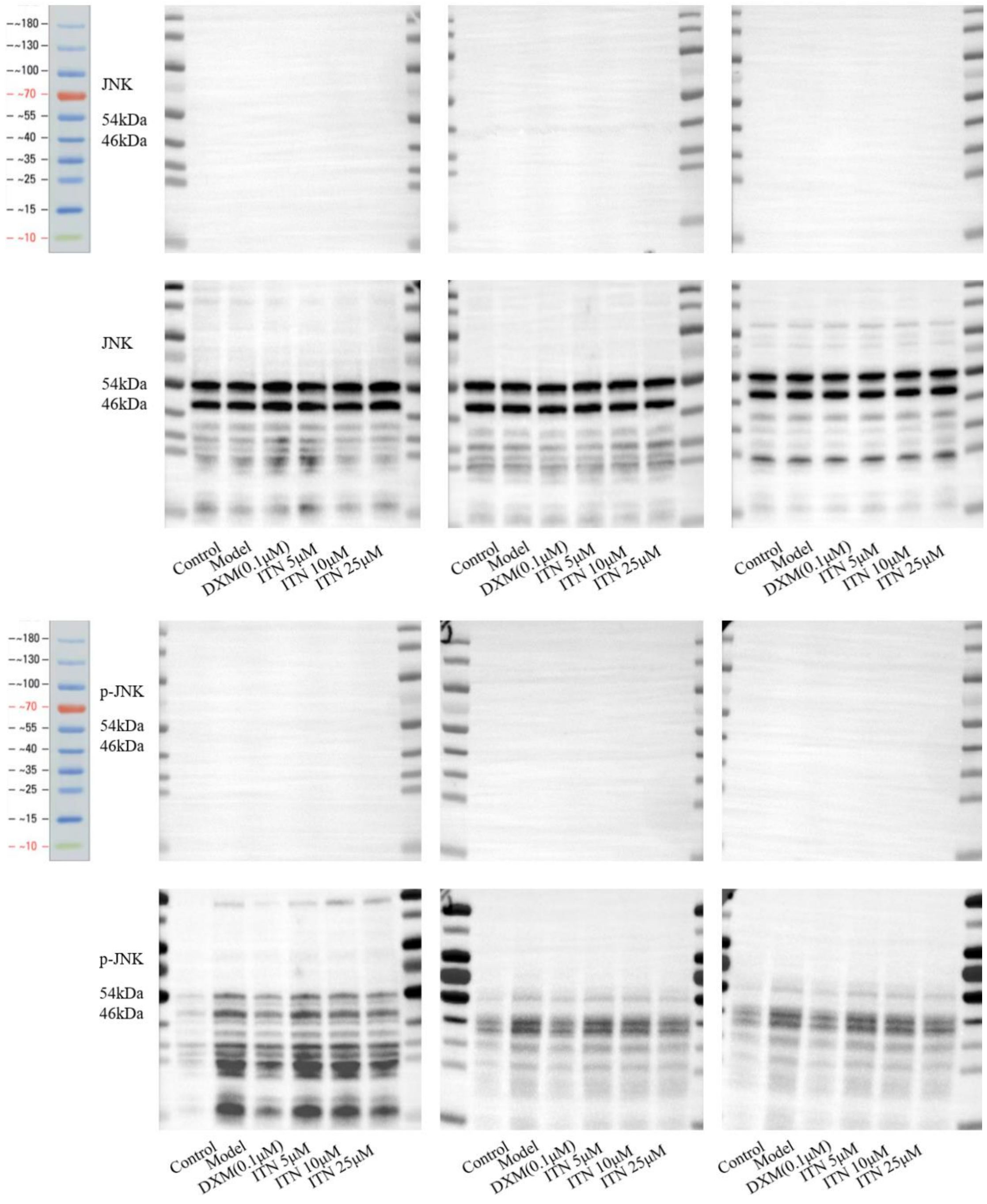
~180
~130
~100
~70
~55
~40
~35
~25
~15
~10
JNK
54kDa
46kDa
JNK
54kDa
46kDa
Control
Model
DXM(0.1μM)
ITN 5μM
ITN 10μM
ITN 25μM
p-JNK
54kDa
46kDa
p-JNK
54kDa
46kDa

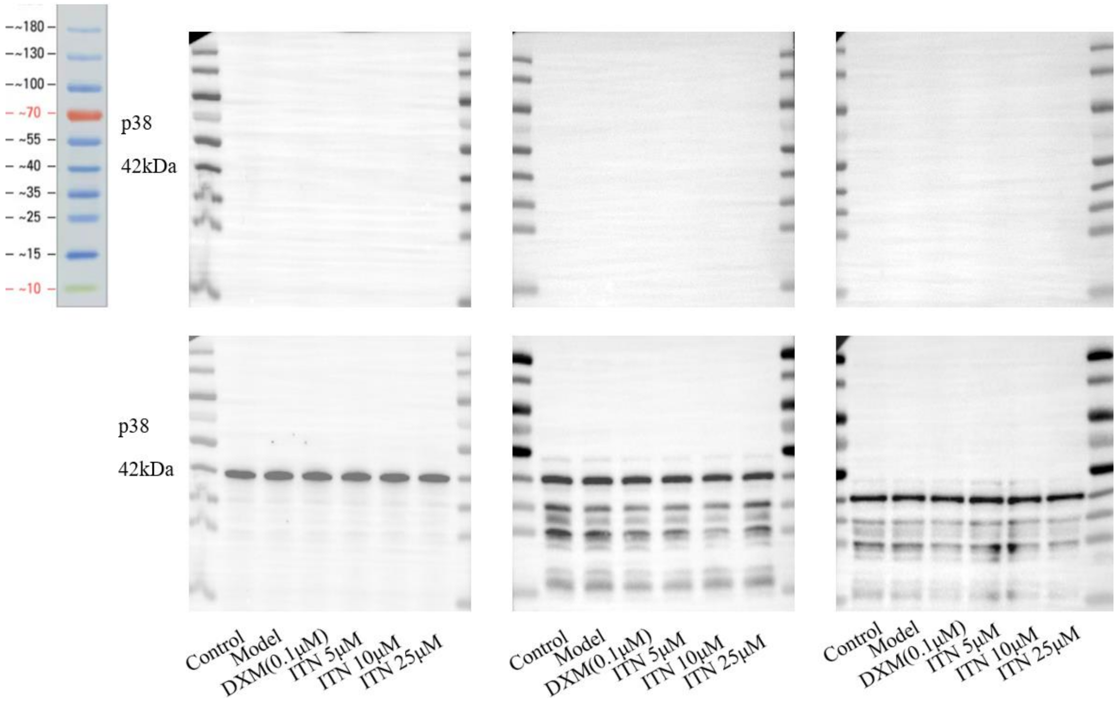
~180
~130
~100
~70
~55
~40
~35
~25
~15
~10
p38
42kDa
p38
42kDa
Control
Model
DXM(0.1μM)
ITN 5μM
ITN 10μM
ITN 25μM

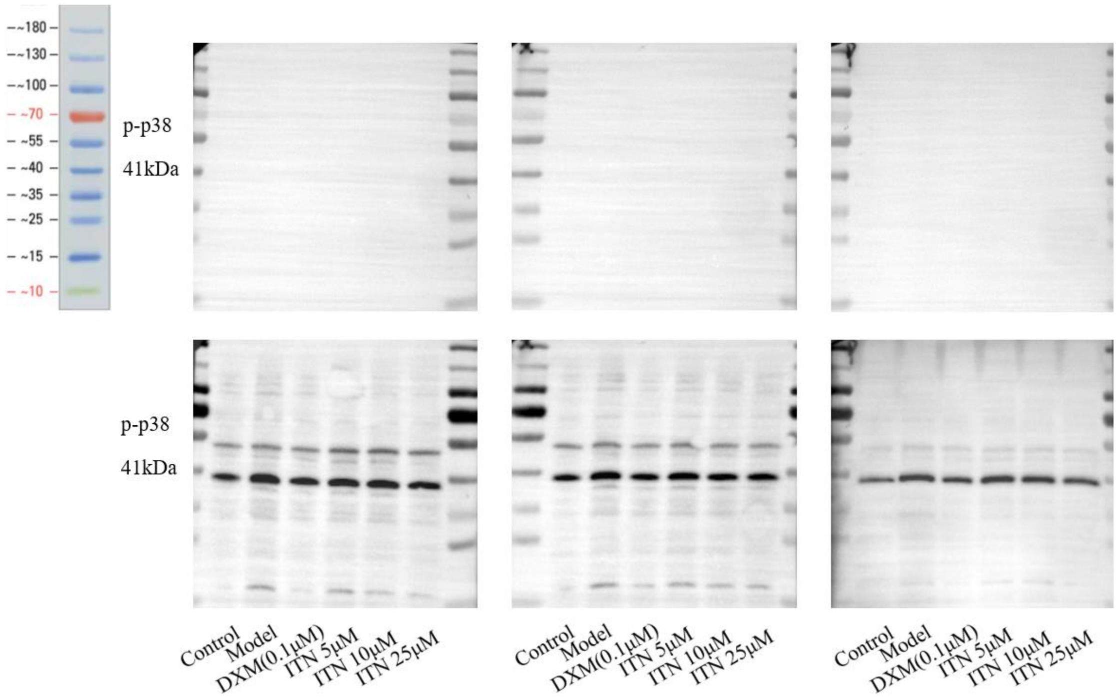
~180
~130
~100
~70
~55
~40
~35
~25
~15
~10
p-p38
41kDa
p-p38
41kDa
Control
Model
DXM(0.1μM)
ITN 5μM
ITN 10μM
ITN 25μM

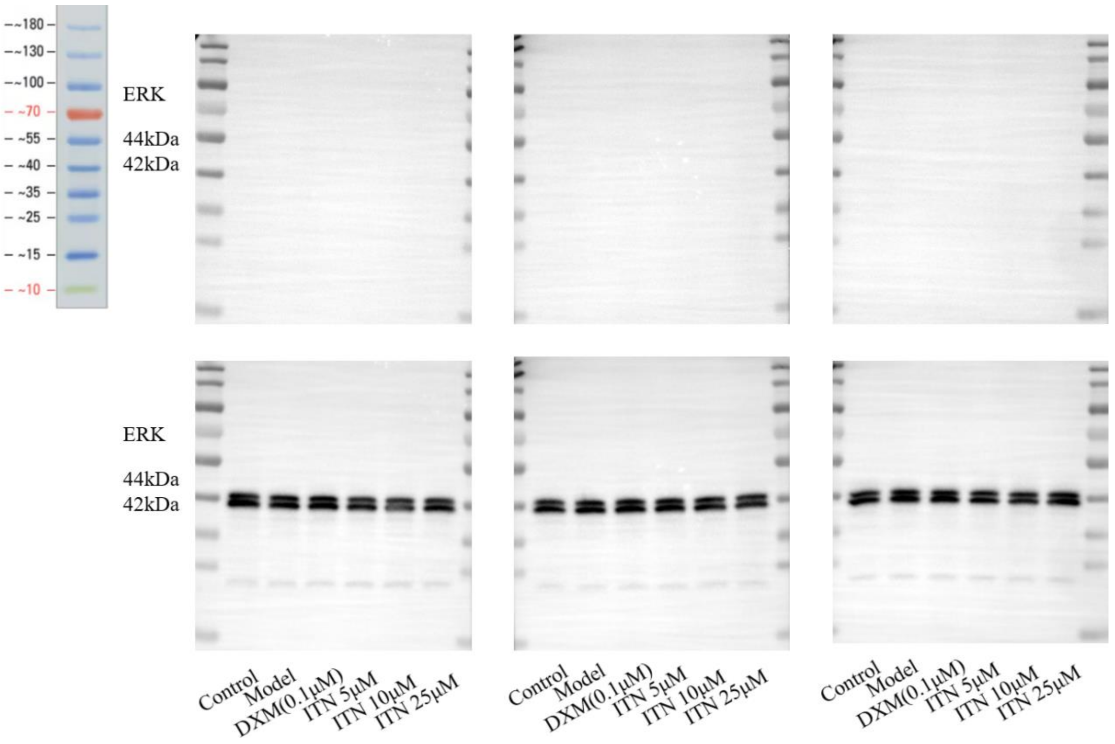
~180
~130
~100
~70
~55
~40
~35
~25
~15
~10
ERK
44kDa
42kDa
ERK
44kDa
42kDa
Control
Model
DXM(0.1μM)
ITN 5μM
ITN 10μM
ITN 25μM
Control
Model
DXM(0.1μM)
ITN 5μM
ITN 10μM
ITN 25μM
Control
Model
DXM(0.1μM)
ITN 5μM
ITN 10μM
ITN 25μM

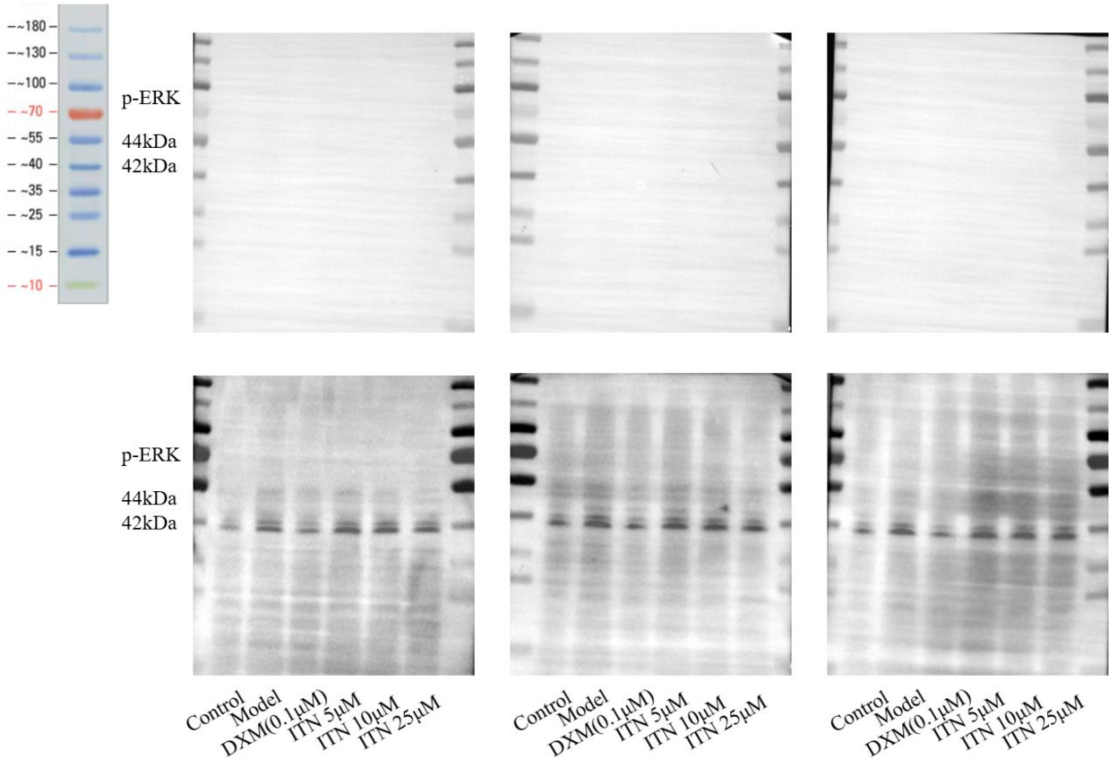
~180
~130
~100
~70
~55
~40
~35
~25
~15
~10
p-ERK
44kDa
42kDa
p-ERK
44kDa
42kDa
Control
Model
DXM(0.1μM)
ITN 5μM
ITN 10μM
ITN 25μM
Control
Model
DXM(0.1μM)
ITN 5μM
ITN 10μM
ITN 25μM
Control
Model
DXM(0.1μM)
ITN 5μM
ITN 10μM
ITN 25μM

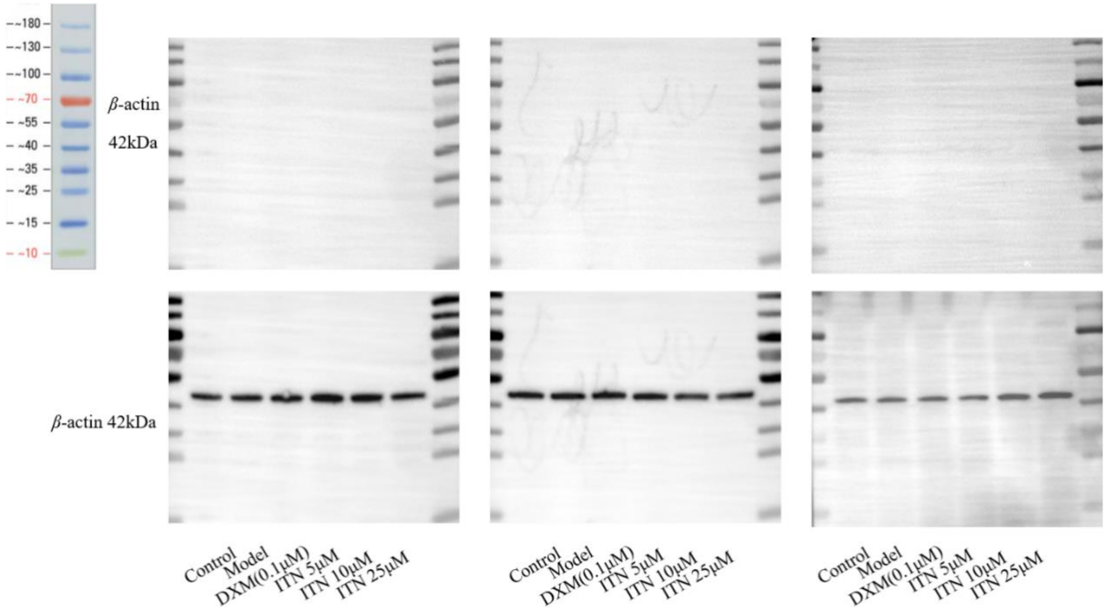

Figure S2. Effects of ITN on MAPKs pathway in LPS irritated A549 cells.

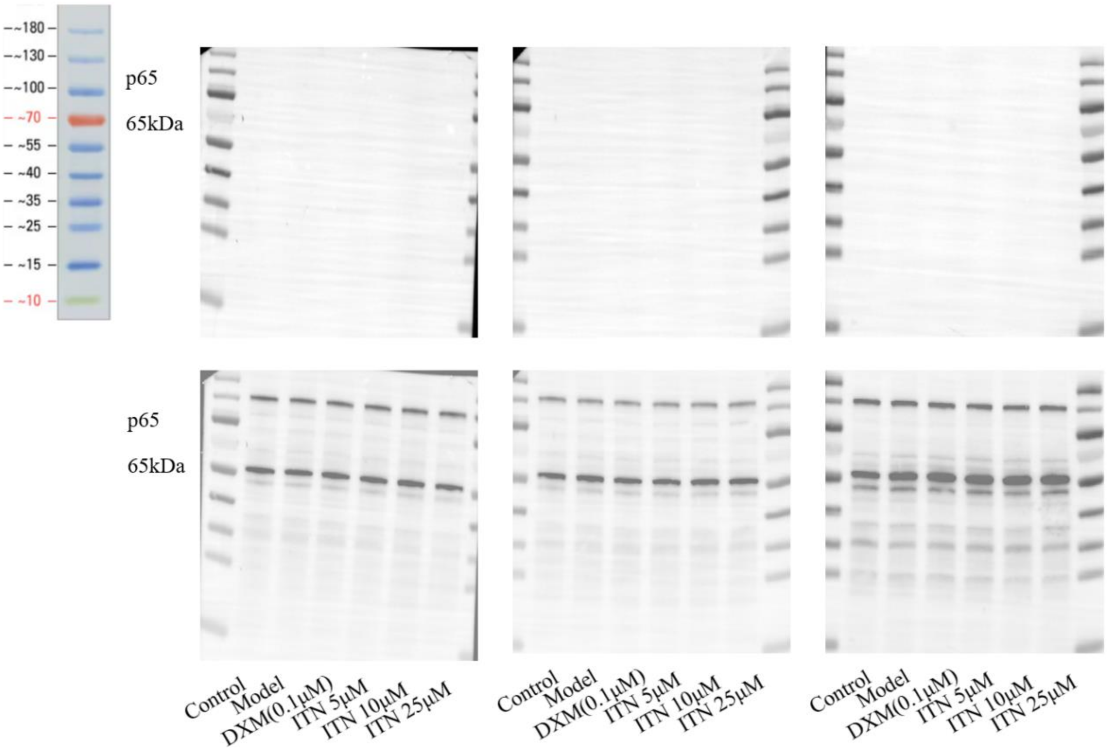

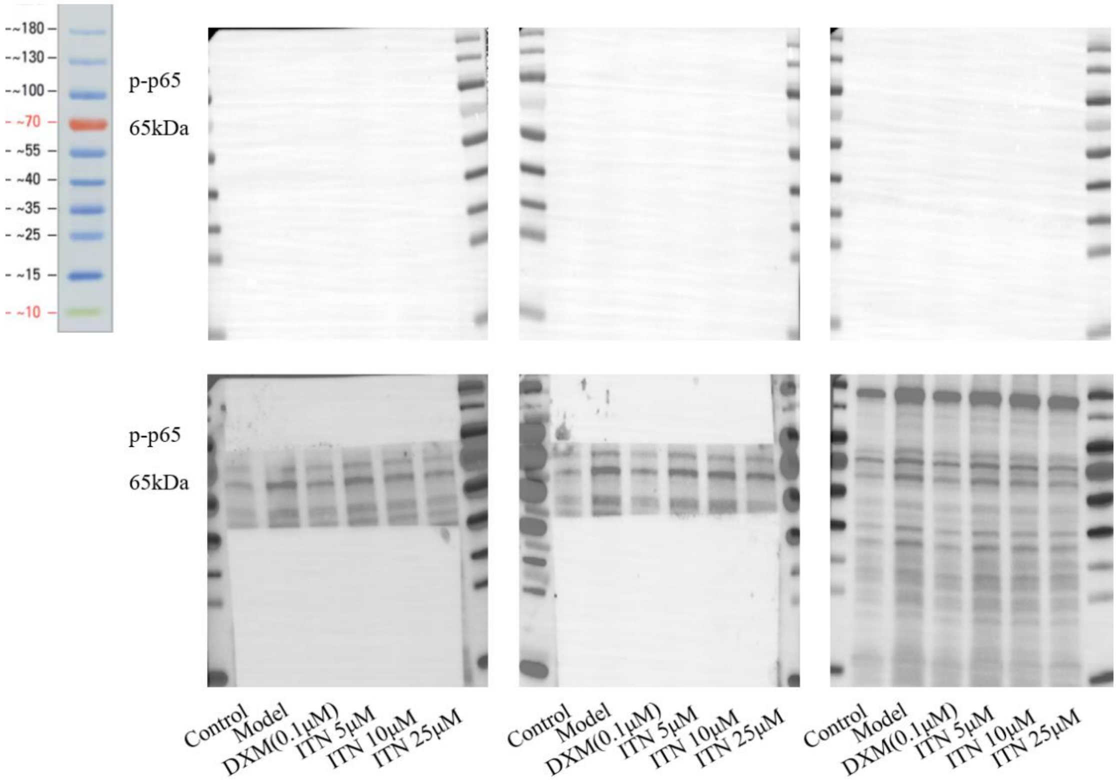
~180
~130
~100
~70
~55
~40
~35
~25
~15
~10
p-p65
65kDa
p-p65
65kDa
Control
Model
DXM(0.1μM)
ITN 5μM
ITN 10μM
ITN 25μM
Control
Model
DXM(0.1μM)
ITN 5μM
ITN 10μM
ITN 25μM
Control
Model
DXM(0.1μM)
ITN 5μM
ITN 10μM
ITN 25μM

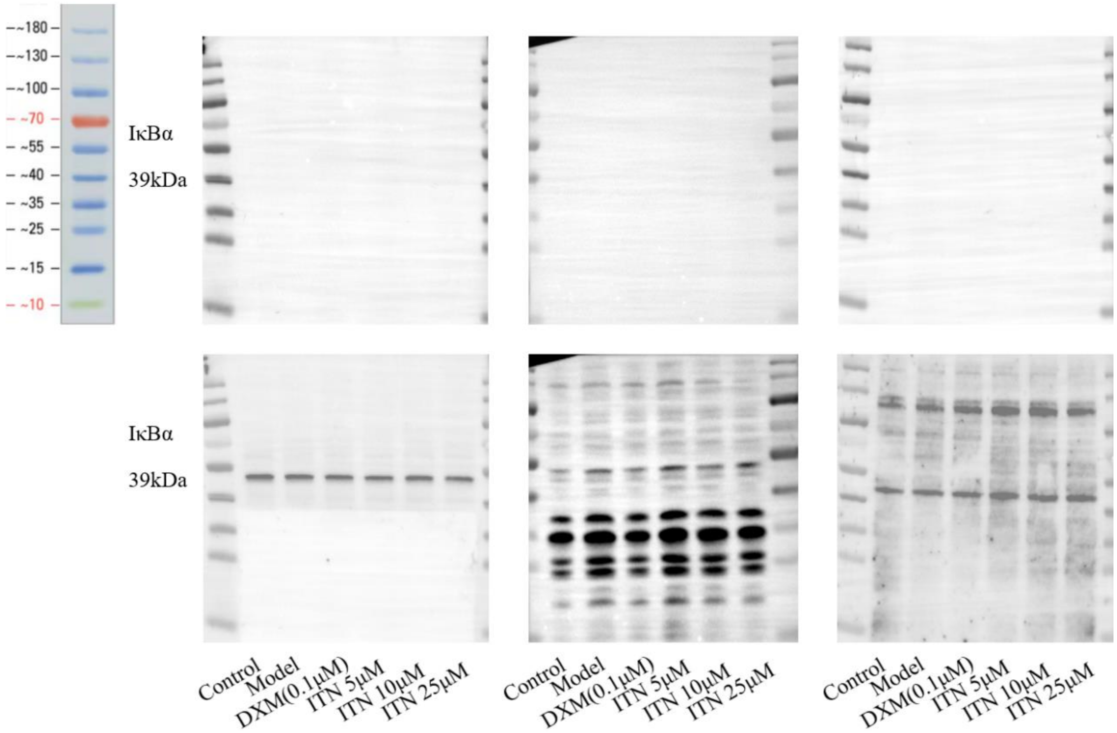
~180
~130
~100
~70
~55
~40
~35
~25
~15
~10
IκBα
39kDa
IκBα
39kDa
Control
Model
DXM(0.1μM)
ITN 5μM
ITN 10μM
ITN 25μM
Control
Model
DXM(0.1μM)
ITN 5μM
ITN 10μM
ITN 25μM
Control
Model
DXM(0.1μM)
ITN 5μM
ITN 10μM
ITN 25μM

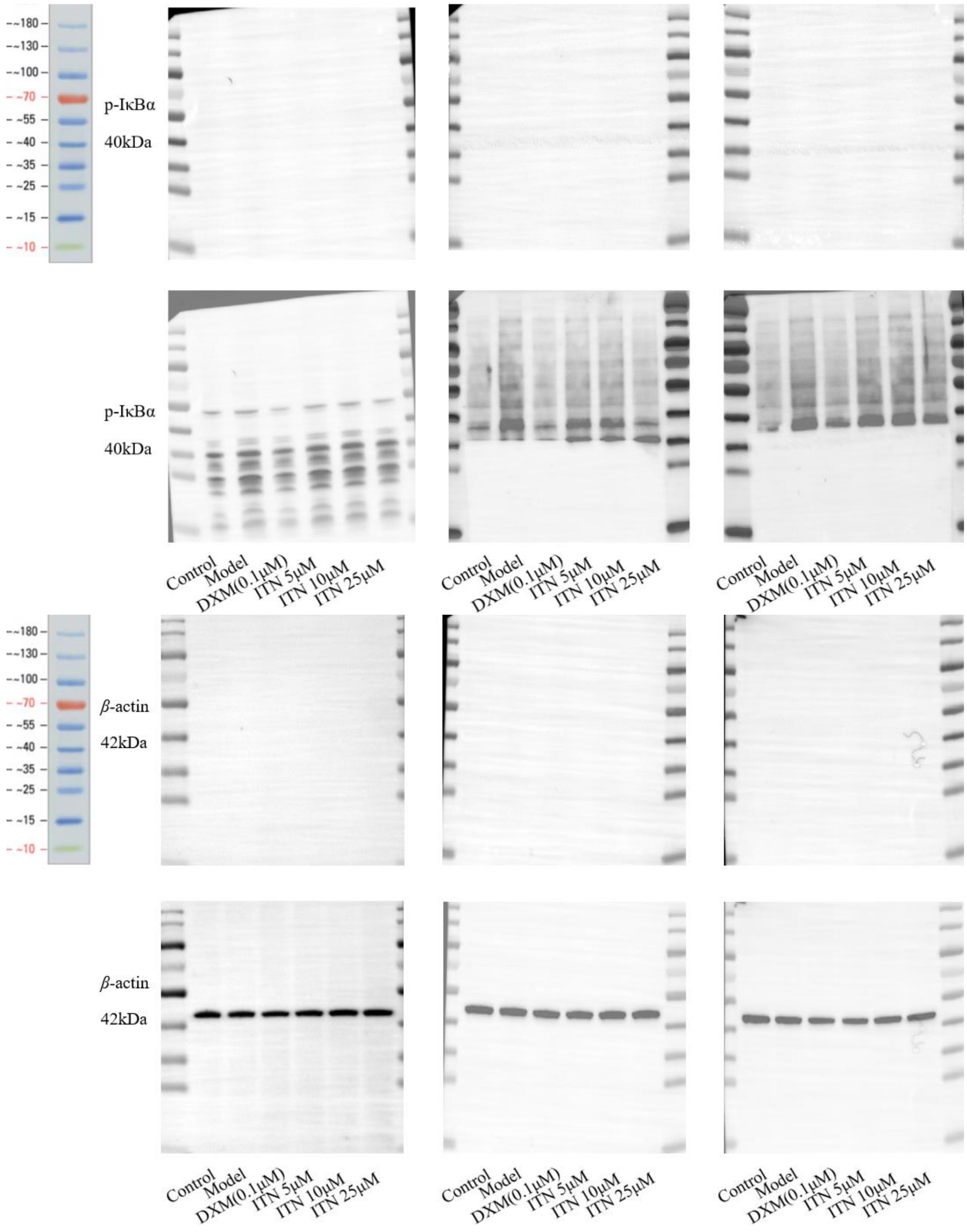

Figure S3. Effects of ITN on NF-κB pathway in LPS irritated A549 cells.
